# Electrophoretic Deposition of WS_2_ Flakes on Nanoholes Arrays—Role of Used Suspension Medium

**DOI:** 10.3390/ma12203286

**Published:** 2019-10-10

**Authors:** Dario Mosconi, Giorgia Giovannini, Nicolò Maccaferri, Michele Serri, Stefano Agnoli, Denis Garoli

**Affiliations:** 1 Dipartimento di Chimica, Università degli Studi di Padova, Via Marzolo 1, 35131 Padova, Italy; dario.mosconi@unipd.it (D.M.); stefano.agnoli@unipd.it (S.A.); 2 EMPA Federal Swiss research Institute, 9014 St. Gallen, Switzerland; giorgia.giovannini@iit.it; 3 Physics and Materials Science Research Unit, University of Luxembourg, L-1511 Luxembourg, Luxembourg; nicolo.maccaferri@uni.lu; 4 Istituto Italiano di Tecnologia, via Morego 30, 16163 Genova, Italy; michele.serri@iit.it

**Keywords:** WS_2_, nanopores, integrated systems, electrophoretic deposition, plasmonics, photoluminescence, Raman spectroscopy

## Abstract

Here we optimized the electrophoretic deposition process for the fabrication of WS_2_ plasmonic nanohole integrated structures. We showed how the conditions used for site-selective deposition influenced the properties of the deposited flakes. In particular, we investigated the effect of different suspension buffers used during the deposition both in the efficiency of the process and in the stability of WS_2_ flakes, which were deposited on an ordered arrays of plasmonic nanostructures. We observed that a proper buffer can significantly facilitate the deposition process, keeping the material stable with respect to oxidation and contamination. Moreover, the integrated plasmonic structures that can be prepared with this process can be applied to enhanced spectroscopies and for the preparation of 2D nanopores.

## 1. Introduction

Over the last decade, two-dimensional (2D) materials have attracted much interest and intensive scientific endeavor has been devoted to their investigation. Initially, graphene was drawing all attention, as indicated by the wide array literature related to the carbon-based material. Nowadays, interest has been broadened to other types of 2D materials like boron nitride (BN); transition metal dichalcogenides (TMDs) such as MoS_2_ and WS_2_; transition metal oxides (TMOs); as well as others including black phosphorous, silicene, etc. [1,2,3,4]. These materials are under deep investigation due to their diverse properties and potential applications spanning from photonics, electronics, biosensing and catalysis [5,6,7]. Among the others, researchers have been particularly interested in exploring the potential of TMDs semiconductors [7]. For instance XS_2_, XSe_2_, etc., exhibit strong light-matter coupling and possess direct band gaps in the infrared and visible spectral regimes, making them interesting for various applications in optics and optoelectronics [6]. However, the control of the optical properties of a XS_2_ layer(s) is still a great challenge and significant efforts are still required. In principle, the active control of XS_2_ properties (such as photoluminescence, strong light–matter interaction, electrochemical activity, etc.) can be obtained by utilizing photonic and plasmonic nanostructures [8,9,10,11,12,13,14]. It is noteworthy that, in order to find application in photonics and plasmonics, the 2D material (e.g., XS_2_ layers) must be integrated with suitable metallic or dielectric nanostructures [13,15,16,17,18]. This can be done by depositing nanoparticles or nanostructures over the 2D layer, a procedure typically done by means of complex and time-consuming lithographies. An alternative approach consists in the deposition of the 2D layer over the nanostructures, but this requires transfer procedures that are quite challenging and time-consuming. 

In this context, we have recently proposed and demonstrated the possibility to control the deposition of single layer MoS_2_ flakes on metallic nanostructures by means of chemical conjugation and electrophoretic deposition (EPD) [19,20]. The EPD method we reported, in particular, enables the deposition of MoS_2_ flakes to any substrates comprising nanoapertures. Moreover, it can selectively decorate nanoholes with flakes with either single or few layers. EPD is a colloidal process in which the particles, having acquired an electric charge in the liquid in which they are suspended, move towards one of the electrodes once an electric field is applied. Usually the electrode corresponds to the substrate on which the deposition of 2D material is desired. EPD has been extensively applied to 2D materials for several applications, such as sensing, Li-ion batteries, anticorrosive coatings, etc. [21,22,23,24]. The kinetics of EPD depends on different parameters. For this study we exploited the alternative EPD approach extensively described in our previous work [20] in which the substrate on which 2D materials are deposited is placed in the middle of the electrophoretic chamber allowing the selective decoration of the nanoholes of the substrate and the reuse of the EPD chamber. Here, theoretical and modeling studies are presented to clarify the mechanisms of EPD, and experimental data related to the EPD of 2D materials (WS_2_) on metallic nanoholes are reported. We investigated electrochemical parameters such as conductivity of the solvents, zeta potential of the flakes, intensity of the electric field, sample concentration, etc. Indeed, we observed during our previous work [20] that the suspension buffer used for the EDP determines the efficacy and efficiency of the site-selective deposition. In this view, we investigated different buffers looking at the kinetics of EPD and at the stability of the material in terms of contamination and oxidation. We observed that the choice of the proper buffer significantly improves the deposition kinetic while maintaining the structural composition of the material. 

We believe that the method here optimized can be used to prepare structures that can find several interesting applications in the research fields based on 2D materials [9,10,11,18,25,26,27,28,29,30]. For example, here we report three different potential applications, such as fabrication of hybrid nanopores, enhanced Raman spectroscopy, and photoluminescence. Numerical simulations are reported to support the observed enhanced light–matter interaction.

## 2. Materials and Methods 

### 2.1. WS_2_ Exfoliation

In a glove-box (water < 1 ppm, O_2_ < 10 ppm), LiBH_4_ (0.130 g, 6 mmol) and WS_2_ (0.496 g, 2 mmol) were ground in a mortar and subsequently transferred in a Schlenk tube, which was then brought outside of the glove-box and connected to a Schlenk line. The sample was heated in a sand bath at 340 °C for 5 days under nitrogen. The so-obtained intercalation compound was added to 300 mL of degassed water in one shot, and the resulting suspension was bath-sonicated for 1 h. In order to remove LiOH, the material was washed three times by centrifugation at 10,000 rpm (23,478× *g*) for 15 min. To remove lighter nanosheets the sample was centrifuged at 8000 rpm for 15 min collecting the solid cake, which was then redispersed in deionized H_2_O and centrifuged at 1000 rpm for 5 min to let the unexfoliated material to settle.

### 2.2. X-ray Photoemission Spectroscopy (XPS)

XPS is performed in a custom designed ultrahigh vacuum (UHV) system comprising a load lock and an analysis chamber equipped with an Omicron electron analyzer (EA125, Scienta Omicron GmbH, Taunusstein, Germany), and a dual anode X-ray source (Omicron DAR 400, Scienta Omicron GmbH, Taunusstein, Germany). The materials to be investigated are drop-cast on a copper support to form a homogeneous thin film. The dried samples are then inserted in the UHV system and left outgassing extensively for a few hours. All XPS measurements are performed under UHV conditions (<10^−8^ mbar) at room temperature using a non-monochromated Mg K_α_ source (h_μ_ = 1253.6 eV) and an electron analyzer pass energy of 20 eV. The binding energy (BE) scale is calibrated using the Au 4f7/2 core levels (BE = 83.8 eV). XPS multipeak analysis (KolXPD software, 1.8.0) was performed using Voigt functions, keeping constant in all the peaks the full width at half maximum (FWHM) and the Gaussian-Lorentzian proportion, which allow to obtain the maximum reliability of the fitting. For the same reason, BEs regarding the same compounds were kept in ±0.1 eV range from one sample to another.

### 2.3. Buffer Preparation

10 mM MES buffer was prepared by dissolving the required amount of 4-morpholineethanesulfonic acid (Sigma, Italy) in DI water. The pH of the solution was adjusted by NaOH (0.1 M) addition until the desired value.

10 mM PBS buffer was prepared following the Seller’s instruction: one tablet was dissolved in 200 mL of DI water yielding 10 mM phosphate buffer, 2.7 mM potassium chloride, 137 mM sodium chloride, pH 7.4, at 25 °C (Sigma). This solution was then diluted with DI water to achieve PBS 1 mM and 0.1 mM.

B-1: Na_2_SO_4_ (Sigma) and NaOH (Sigma) were solubilized in DI water reaching respectively 1.67 mM and 1 mM as final concertation. The pH of this solution was found to be 10.8.

### 2.4. Measurement of Dynamics Light Scattering of WS_2_ Flakes

DLS experiments were performed using a Malvern Zetasizer nano ZS (Malvern Instruments, Malvern, UK) and the measurements were evaluated using Zetasizer software (version 3.3). The electrophoretic mobility was evaluated using Henry’s equation following the assumptions used in [20]. Data are calculated as the average of three measurements (n = 3) ± SD. Samples were measured at 25 °C in disposable folded capillary cells (DTS1070) in aqueous dispersants.

### 2.5. FEM Simulation of WS_2_ Flakes Integrated over Plasmonic Nanostructures

The plasmonic properties of the structure have been investigated by means of finite element method (FEM) simulations using an RF module in Comsol Multiphysics, taking into account the geometries that were actually fabricated. A hole of 100 nm in diameter in a 100 nm thick Si_3_N_4_ membrane with a 50 nm thick Au layer has been considered with a top single layer of WS_2_. The refractive indices of Au and WS_2_ were taken from the works of Rakic et al. [31] and Zhang et al. [32]. A single nanostructure was considered by setting the unit cell to be 400 nm wide in both the x- and y-directions, with perfect matching layers (150 nm thick) at the borders. A linearly polarized incident plane wave was assumed to impinge on the structure from the air side.

### 2.6. Fabrication of Plasmonic Nanoholes

For the fabrication of the metallic nanoholes we used a Si_3_N_4_ membrane (100 nm thick) substrate prepared on a Silicon chip. The 2D holes were milled by means of FIB with a voltage of 30 keV and a current of 80 pA. After milling, a thin layer of gold, ca. 50 nm, was deposited on the top side of the membrane.

### 2.7. Electrophoretic Deposition

EPD has been performed following the method illustrate in details by Mosconi et al. [20]. In summary, the substrate is not used as electrode, on the contrary, the nanoholes present in it allow for the through-flow of ions, whilst also representing a barrier to the WS_2_ flakes, which cannot pass due to their size and are deposited. The metallic nanoholes array prepared on a Si_3_N_4_ membrane is first cleaned in oxygen plasma for 60 s to facilitate the deposition. The sample (at 0 V potential) is placed in the microfluidic chamber and the chamber sides are filled with WS_2_ suspended in different suspension buffers and the buffer alone, respectively (reported below). A suitable voltage for the required deposition thickness is then applied for a few minutes to allow for electrophoretic deposition. The chamber is then opened, and the deposited final sample is rinsed with EtOH.

### 2.8. Optical Spectroscopies—Raman and Photoluminesce

Raman spectroscopy has been performed by using a Renishaw InVia Microscope Raman system with a 50 × 0.95 NA objective. An excitation wavelength of 532 nm has been used. The signal has been collected with a spectral resolution of 2.5 cm^−1^ and an integration time of 1 s. The system was calibrated by using the intensity of the standard peak at 520 cm^−1^ from a silicon substrate. The same microscope has been used as spectrometer to measure the emitted photoluminescence from the decorated nanoholes array.

## 3. Results and Discussion

In this work, three different suspension buffers at different pHs and concentrations were tested and compared with water, and solvent in which WS_2_ was initially suspended. Among the others, we chose MES and PBS since are buffers commonly used for several bio-applications and therefore easily accessible. These two buffers have also different properties. Whereas PBS readily forms coordination complexes in presence of metals, MES is used as non-coordinating buffer in chemistry involving metal ions. Furthermore, we used a third buffer (B-1) with a relatively low salt concentration and basic pH. The formulations of these buffers are reported in Table 1. MES was prepared at different pHs (from 3 to 8) adjusted by addition of NaOH (0.1 M), PBS was tested at different concentrations (10, 1 and 0.1 mM), whereas B-1 was prepared solubilizing Na_2_SO_4_ NaOH reaching 1.67 and 1 mM as final concentration. The so-achieved buffer pH 10.8 was measured.

In particular, we investigated two key aspects which are related to the suspension buffer used: (i) the zeta-potential and the conductivity of the flakes’ suspensions; and (ii) the properties of the WS_2_ after the EDP process.

### 3.1. Evaluation of EPD Parameters

One of the parameters that determines the EPD deposition is the net mean charge of the WS_2_ in the selected buffer. For this reason, a detailed analysis of the WS_2_ flakes electrophoretic mobility (EM) and zeta potential (ζ) is required.

WS_2_ flakes are characterized by a negative surface charge (−46.87 ± 0.71 mV) when suspended in deionized (DI) water as indicated by the ζ value measured by dynamic light scattering (DLS). ζ is also known as electrokinetic, which is the potential at the surface–fluid interface of a colloid moving under electric field. Indeed, once suspended in a buffer, ions of opposite charge will be absorbed on the WS_2_ surface forming the so called electric double layer (EDL) which determines the electrical mobility of the flakes in suspension under electric field and that correspond to the ζ measured by DLS. The surface charge of WS_2_ flakes was tested in the different buffers and the obtained data were considered analyzing the experimental EPD kinetics. The buffers tested have different ionic strength (IS) and different pH. In particular, IS for MES was calculated 0.005, 0.0033 was found for B-1 while the IS for PBS buffers were 0.021, 0.0021, and 0.00021 for respectively PBS at 10, 1, and 0.1 mM.

The values of ζ, EM and conductivity measured by DLS are presented in Table 2.

As noticed from Table 2, the ζ and EM are proportional between each other. DLS software indeed converts the EM measured for a colloidal suspension in the corresponding ζ using Henry equation, where *ε* and *η* are respectively the dielectric constant and viscosity of the solvent. Henry function, *f*(*kα*), instead correlates parameters related to the suspended colloidal—i.e., the radius of the colloids (*α*)—and to the environment (*k*).

(1) $$\mu_{e}=\frac{2\varepsilon zf\left(k\alpha \right)}{3\eta }$$

The variable *f*(*kα*) is inversely proportional to the temperature (*T*) and directly proportional to the ionic strength (*I*) of the solvent as

(2) $$\frac{1}{k}=\sqrt{\frac{\in_{0}\varepsilon Tk_{b}}{2000e^{2}IN}}$$

Two values are generally used as approximation of the *f*(*kα*): 1.5 and 1. For colloidal aqueous suspensions, with moderated electrolyte concentration (salt > 1 mM) *f*(*kα*) is 1.5, known as the Smoluchowski approximation.

ζ measured for the WS_2_ flakes is strongly related to the IS and pH of the buffer in which are suspended. In the case of PBS for instance, the ζ becomes more negative with the increase of IS (from −27.40 ± 0.62 mV for PBS 0.1 mM—IS 0.00021, to −36.87 ± 1.37 mV for PBS 10 mM—IS 0.021). Less difference were observed using MES at the same concentrations (10 mM, IS 0.005) but different pHs, indeed ζ decreased from −27.07 ± 1.56 to −33.07 ± 0.62 mV when measured at pH 3 and 8, respectively. Importantly, the ionic strength of the buffer strongly interferes on the conductivity of the suspension. In particular, the decrease in conductivity is proportional to the decrease of the IS: 13.33 mS/cm^−1^ was the conductivity measured for PBS 10 mM (IS 0.021) which dropped to 1.73 and 0.16 mS/cm for respectively 1 mM (IS 0.0021) and 0.1 mM (IS 0.00021) PBS buffers. On the other hand, less difference was observed in terms of conductivity varying the pH of the solvent and keeping the ion concentration constant: the lowest and higher value measured were 0.04 and 0.79 mS cm^−1^ achieved respectively for the flakes suspension in MES pH 4 and MES pH 8, respectively. The ζ measured for the WS_2_ flakes suspended in B-1 was highly negative (−37.57 ± 0.46 mV) likely due to the basic pH of such buffer (pH 10.8), whereas the IS (0.0033) leads to a moderate conductivity measured for the suspension (0.48 mS cm^−1^). Worth of notice is that, for all buffer tested, the conductivity was improved compared to DI water, for which 0.001 mS cm^−1^ was the value found.

### 3.2. Site Selctive EPD

**Figure 1:**
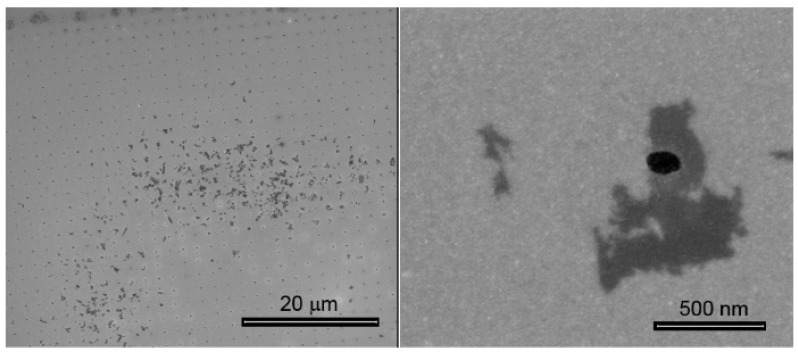
EPD of WS_2_ suspended in H_2_O.

Figure 1 reports the result of EPD performed on WS_2_ flakes dispersed in DI water. The process has been done applying a voltage of 25 V for 5 min. At the end of the process, the array of metallic holes was decorated but with low efficiency since the flakes were deposited with unsatisfied uniformity on the array. This result confirmed our previous findings achieved with MoS_2_ [20].

**Figure 2:**
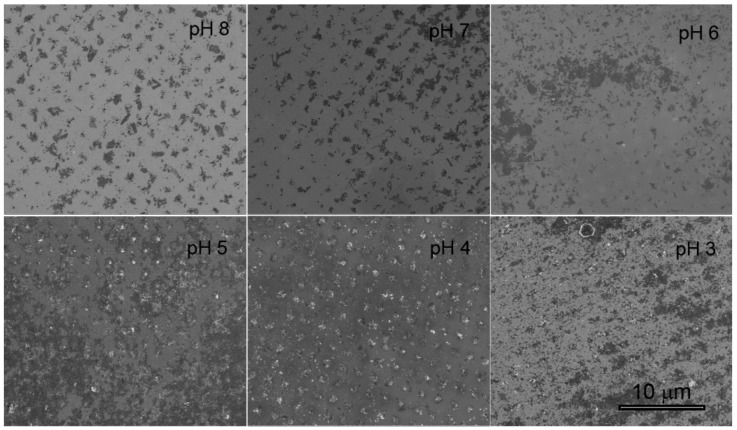
EPD of WS_2_ suspended in MEF with different pH.

**Figure 3:**
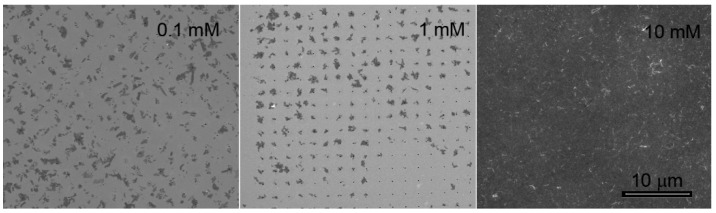
EPD of WS_2_ suspended in PBS at different concentrations.

**Figure 4:**
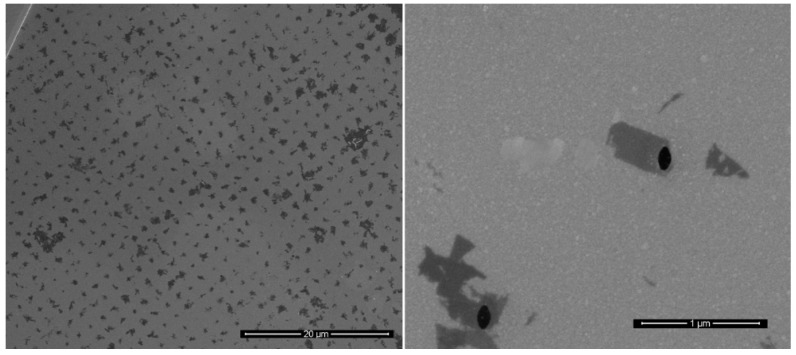
EPD of WS_2_ suspended in B-1.

The reason for this unsatisfactory result was probably related to the low conductivity of the sample (0.001 mS cm^−1^) due to which high voltage (25 V, 5 min) were required for the deposition despite the highly negative ζ (−46.87 ± 0.71 mV) of WS_2_ flakes suspended in DI water. It was therefore concluded that ζ of the suspended flakes should be considered along with the conductivity of the sample in defining the best conditions for an efficient EPD. In order to improve the kinetics of deposition, DI water was replaced with aqueous solution of different salts (MES, PBS, and B-1) at different concentration and pHs. Figure 2, Figure 3 and Figure 4 illustrate the results obtained with MES, PBS, and B-1 by applying a significantly lower voltage equal to 3 V for 5 min, compared with the 25 V required by the experiment in water. It is important to note that with this low potential it was not possible to achieve an EPD in DI water.

Figure 2 shows the EDP of WS_2_ suspended in MES 10 mM at different pHs. These results pointed out the importance of considering both ζ and conductivity in choosing environmental parameters. Indeed, in the case of pH 3 and pH 6 in which ζ was less negative (−27.07 ± 1.56 mV and −28.87 ± 0.66 mV respectively), despite the relatively good conductivity (0.26 and 0.29 mS cm^−1^), it was not sufficient to ensure a proper deposition on the array. With MES pH 7 the increment in conductivity to 0.62 mS cm^−1^ was enough to achieve a better deposition even though the ζ was similar to the previous cases (−28.90 ± 0.43 mV). On the contrary, in spite of the low conductivity measured for MES pH 4 (0.04 mS cm^−1^) the deposition achieved was acceptable likely due to the lower ζ of WS^2^ in such buffer (−31.50 ± 0.57 mV).

EDP results using PBS at different concentrations and therefore different ISs (Figure 3) suggested the importance of tuning all parameters in order to achieve EDP of 2D material with good efficiency and efficacy. As noticeable indeed, the highly negative ζ (−36.87 ± 1.37 mV) and high conductivity (13.33 mS cm^−1^) of WS_2_ in PBS 10 mM, IS 0.021 lead to a massive and disordered deposition of flakes on the array, which were likely ascribed to the aggregation of flakes at this concentration. Better results were observed instead using lower concentrations of PBS. In particular, using PBS 1 mM (IS: 0.0021; ζ: −34.57 ± 1.29 mV; conductivity: 1.73 mS cm^−1^), WS_2_ flakes were selectively deposited in one the nanoholes of the array with very limited unspecific deposition.

The combination of ζ and conductivity found using B-1 as buffer (−37.57 ± 0.46 mV; 0.48 mS cm^−1^) enabled a reproducible and ordered deposition of thin flakes over the hole array (Figure 4).

### 3.3. XPS Analysis

**Figure 5:**
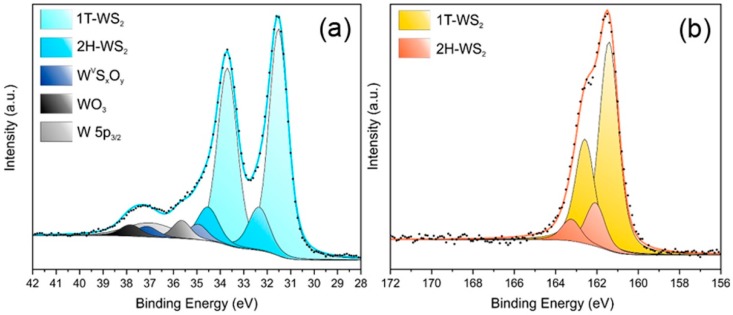
XPS spectra of exfoliated WS_2_ W 4f (**a**) and S 2p (**b**) photoemission lines.

XPS has been used as a tool to evaluate the effects of the used buffer on the contamination/oxidation of the WS_2_ flakes. As starting point, exfoliated WS_2_ nanosheets simply suspended in DI water were analyzed as a reference. The separation into chemically shifted components of the W 4f and S 2p photoemission spectra is reported on Figure 5. The metallic WS_2_-1T phase typically shows a W 4f peak maximum at 31.5 eV, whereas the component associated with the 2H polymorph is centered at 32.3 eV. On the high BE side of the W 4f spectrum, it is possible to identify the presence of WO_3_, characterized by the W 4f_7/2_ peak at 35.7 eV. However, to achieve a satisfying fit, a fourth component relative to oxidized W is needed: the resulting BE is intermediate between W^VI^O_3_ (35.7 eV) and the value reported in the literature for W^IV^O_2_ (32.8 eV) [33], therefore we can hypothesize the presence of an intermediate oxysulfide phase [34]. The analysis of the S 2p peak follows the same rationale: we can clearly identify two doublets with the maxima at 161.4 eV and 162.1 eV that can be ascribed to the S 2p_3/2_ peak of the 1T and 2H phase, respectively. In terms of atomic percentage, the ratio between the 1T and 2H is about 4.8 as deduced from the spectra of both the S 2p and W 4f core levels.

Table 3 reports the composition of the pristine WS_2_ nanosheets dispersed in DI water in detail. Whereas Figure 6 shows the chemical composition of the the WS_2_ nanosheets as a consequence of the dispersion into the media utilized in EPD process, as deduced by the multipeak analysis of the W 4f core levels (detailed XPS spectra and analyses are reported in Figures S1–S10 and Tables S1–S10 (Supplementary Materials)).

**Figure 6:**
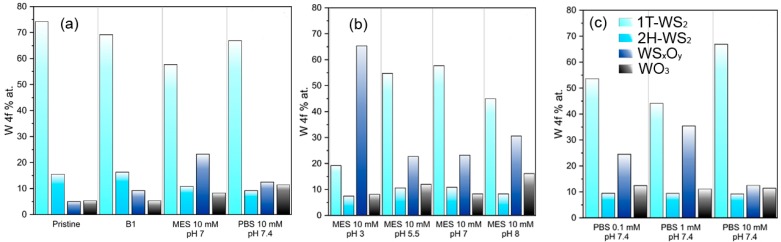
XPS derived composition of WS_2_ in different buffers (where Pristine represents DI H_2_O) (**a**), in different pH MES-based buffers (**b**), and in PBS buffers with increasing IS (**c**).

Comparing the material after the interaction with different media (Figure 6a), it can be seen that the solvent B-1 better preserves the properties of the pristine material. Actually, the sample shows only a very slight oxidation of the 1T phase to W(V), probably as a consequence of the hydrolysis of W-S bonds by hydroxyl ions. Among the tested MES samples (Figure 6b), the one at neutral pH maintains most of the pristine structure, even if a more marked oxidation was observed compared to B-1. The conversion was observed equally for the 2H and 1T phases, which were mainly converted to W(V). PBS 10 mM at pH 7.4, compared to MES, was preserved in a better way the 1T phase, but it is more prone to oxidize to W (VI).

From XPS analysis on MES-based samples, we could observe that MES molecule can interact chemically with WS_2_, generating oxysulfides. At acid pH this action is much faster, since about 75% of pristine 1T-WS_2_ was converted to oxysulfides. This could be due to the precipitation of MEs’ molecules in acidic environment on the deposited 2D material or it could indicate acid catalyzed hydrolysis. The effect is then attenuated increasing the pH, in particular at neutral, where the material is substantially preserved. Going to slightly basic pH values, the material got more damaged and the oxidation to WO_3_ doubled with respect to sample treated at pH 7. Clearly, the different surface chemistry affects the EPD process as well, so it is not surprising that the pH 7 sample allows to obtain the most reproducibile depositions.

Regarding samples prepared using PBS at different concentrations, we could not observe a distinct trend in material preservation, since the oxidation process reaches its maximum at 1 mM concentration, while PBS 10 mM is among the best media for preserving the WS_2_ structure. However, cross-checking with SEM analysis of EPD from PBS 10 mM (Figure 4), we observed a very high deposition rate, which can be ascribed to the high salt concentration that induce the precipitation of the dispersed flakes. Therefore, the aggregation of WS_2_ flakes may protect the material surface from the oxidative interaction with medium, which is not observed in concentration up to 1 mM. Moreover, PBS 1 mM allowed to obtain stable suspensions of non-aggregated flakes (i.e., more exposed to chemical interactions), resulting, consequently, in better quality depositions.

### 3.4. Examples of Applications

As mentioned, TMDs and WS_2_ in particular, find significant application in several fields, from catalysis, to photonics and optoelectronics. The presented EPD protocol can be applied to prepare integrated (hybrid) plasmonic nanostructures enabling light–matter interaction studies, such for instance surface enhanced Raman spectroscopy (SERS) and photoluminescence (PL). Moreover, integrated 2D material-plasmonic nanostructures can find application in nanopore technology for single molecule detection [35] and an easy method for nanopores fabrication in 2D layers has been recently proposed by our group [19]. In these regards, here we report three examples where the EPD of WS_2_ in B-1 suspension medium has been used for: (i) the preparation of an hybrid 2D material-plasmonic nanopore; (ii) the SERS from the array of nanoholes; (iii) the enhanced PL emitted from the nanoholes array.

**Figure 7:**
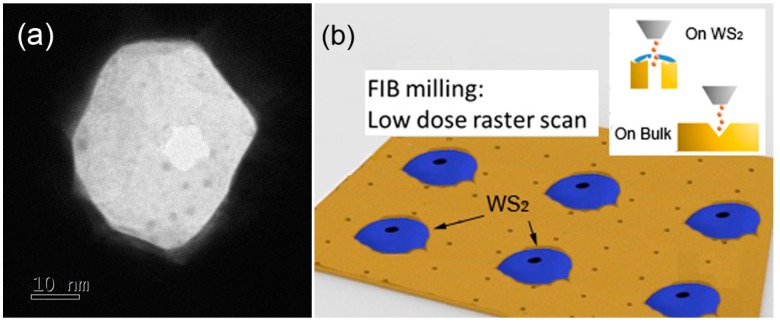
(**a**) TEM micrograph of the nanopore prepared into the 2D layer; (**b**) schematic illustration of the FIB milling over the array.

A sub 10 nm pore has been prepared by means of focused ion beam (FIB) serial milling following the procedure reported in details in [19] (Figure 7a). In summary, the array of metallic holes decorated with WS_2_ flakes has been exposed with Ga^2+^ ions using a current of 24 pA (30 KV of acceleration voltage) with a single raster scan. The use of pitch slightly larger with respect to the nanohole diameter ensures the single hole drilling (Figure 7b). The preparation of this hybrid pore enables a significant electromagnetic field confinement that will be discussed in the next Section 3.5.

**Figure 8:**
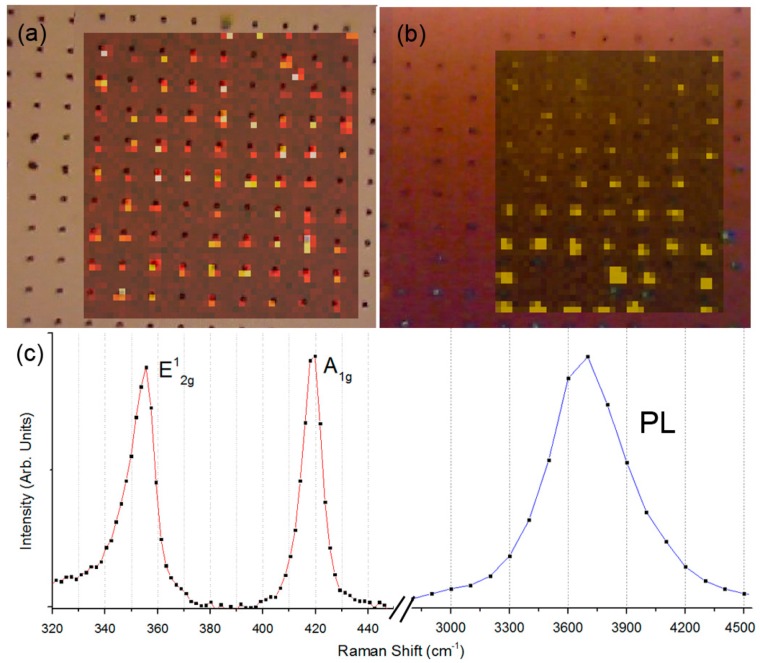
(**a**) Raman Map, integrated on the A_1g_ mode at 410 cm^−1^; (**b**) PL map—excitation at 532 nm—emission integrated between 3000 and 4000 cm^−1^; (**c**) Example of collected spectrum.

Raman spectroscopy, and in particular a Raman map that consider the intensity at 410 cm^−1^ (wavelength shift corresponding to A_1g_ mode), can be used to demonstrate the site-selective deposition and eventually to evaluate the thickness of the deposited flakes [36]. Figure 8a reports an example of the result achieved by this measurement on an EPD samples. The two main features in the Raman spectrum correspond to the E^1^_2g_(Г) and A_1g_(Г) modes (Figure 8c). The relative intensity between the two peaks suggested a few layers (n = 2) deposition [36], even if we demonstrated that EPD can enables single layer deposition [20]. The analyses performed with transmission electron microscope (TEM) (as in Figure 7a) showed that the deposited flakes are not uniform in thickness. As extensively reported in literature [37], exfoliated WS_2_ flakes can present area with one layer (1 L), 2 L, or more. This determines also the intensity of PL emitted from the ordered array of flakes. Figure 8b reports the map obtained integrating the emitted spectrum in the range between 3000 and 4000 cm^−1^, corresponding to 633–675 nm [38]. The PL measured is low even though, the sample was previously annealed for 2 h at 200 °C in vacuum in order to promote the phase transition between 1T to 2H [39]. The low PL signals were expected. This is due to the low number of single layer areas and to the partially metallic phase of the material. However, since we were able to detect PL only from flakes integrated with plasmonic nanohole, this result proves the advantage achievable, integrating a 2D material with a plasmonic nanohole.

### 3.5. Numerical Simulations—Enhanced Electromagnetic Field Confinement

**Figure 9:**
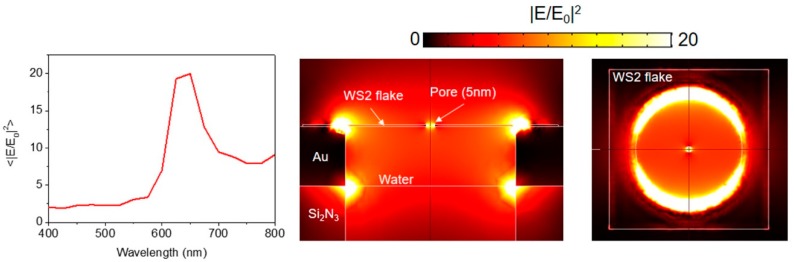
Finite element method (FEM) simulations of the investigated structures covered with one mono-layer of WS_2_ (simulations performed at excitation wavelength 633 nm).

Computer simulations (see Methods for details) can be used to better evaluate potential enhancement effects. In particular, we considered a gold nanohole covered with a single layer WS_2_ flake. Into the 2D layer we designed a pore with a radius of 5 nm. Figure 9 illustrates the obtained field intensity confinement/enhancement that can be achieved. As noticible, the electromagnetic field intensity is highly confined inside the nanohole thanks to the high refractive index of the WS_2_. This, as illustrated in our Raman and photoluminesce experiments, enables enhanced light–matter interaction.

## 4. Conclusions

In the presented work, we proved the importance of considering the properties of both colloidal material and solvent in order to achieve an efficient and controlled EDP of 2D materials on nanoholes. Our results showed that both ζ and conductivity of the 2D material in suspension should be considered in choosing the suitable buffer for EDP. Good results were observed using buffer B-1 with which WS_2_ flakes were deposited in orderly manner over the nanoholes of the array without alteration of their features. Indeed, we showed that the presence of salts in the buffers can interfere with the physical properties of the material likely due to salt or flake precipitation. The final WS_2_ integrated nanostructure can find interesting applications in enhanced light–matter interaction such as PL and Raman as we suggested in this work. Finally, theoretical analysis confirmed that our fabrication method can be applied to prepare plasmonic nanopores for potential single molecule studies.

## Figures and Tables

**Table 1:** Formulation of suspension buffers

Name	Formulation
MES	4-morpholineethanesulfonic acid (MES) 10 mM
PBS	10 mM phosphate buffer. 2.7 mM KCl, 137 mM NaCl.
B-1	1.67 mM Na_2_SO_4_, 1 mM NaOH pH 10.8

**Table 2:** DLS analyses.

MES 10 mM	ζ (mV)		EM (m^2^ s^−1^ V^−1^)		Conductivity (mS cm^−1^)	
	Average	SD±	Average	SD±	Average	SD±
pH 3	−27.07	1.56	−2.12	0.12	0.26	0.00
pH 4	−31.50	0.57	−2.47	0.05	0.04	0.00
pH 5	−25.53	2.93	−2.00	0.23	0.12	0.00
pH 6	−28.87	0.66	−2.26	0.05	0.29	0.00
pH 7	−28.90	0.43	−2.27	0.03	0.62	0.01
pH 8	−33.07	0.62	−2.59	0.05	0.79	0.01
**PBS pH 6.5**						
10 mM	−36.87	1.37	−2.89	0.11	13.33	0.33
1 mM	−34.57	1.29	−2.71	0.10	1.73	0.06
0.1 mM	−27.40	0.62	−2.15	0.05	0.16	0.00
**Other**						
B-1	−37.57	0.46	−2.95	0.04	0.48	0.01
DI Water	−46.87	0.71	−3.67	0.06	0.01	0.01

**Table 3:** XPS of pristine WS_2_.

W 4f			S 2p		
Species	BE (eV)	% at.	Species	BE (eV)	% at.
*1T-WS_2_*	31.5	74.2	*1T-WS_2_*	161.4	82.8
*2H-WS_2_*	32.3	15.5	*2H-WS_2_*	162.1	17.2
*W^V^S_x_O_y_*	34.9	5.0	SO_x_	-	-
*WO_3_*	35.7	5.3			

## Supplementary Materials

The following are available online at https://www.mdpi.com/1996-1944/12/20/3286/s1, Figure S1: W 4f (left) and S 2p (right) core levels of commercial WS_2_. Figure S2: W 4f (left) and S 2p (right) core levels of Pristine 1T-WS_2_. Figure S3: W 4f (left) and S 2p (right) core levels of 1T-WS_2_ deposited from B-1. Figure S4: W 4f (left) and S 2p (right) core levels of 1T-WS_2_ deposited from MES 10 mM-pH 3. Figure S5: W 4f (left) and S 2p (right) core levels of 1T-WS_2_ deposited from MES 10 mM-pH 5. Figure S6: W 4f (left) and S 2p (right) core levels of 1T-WS_2_ deposited from MES 10 mM-pH 7. Figure S7: W 4f (left) and S 2p (right) core levels of 1T-WS_2_ deposited from MES 10 mM-pH 8. Figure S8: W 4f (left) and S 2p (right) core levels of 1T-WS_2_ deposited from PBS 10 mM-pH 7.4. Figure S9: W 4f (left) and S 2p (right) core levels of 1T-WS_2_ deposited from PBS 1 mM-pH 7.4. Figure S10: W 4f (left) and S 2p (right) core levels of 1T-WS_2_ deposited from PBS 0.1 mM-pH 7.4. Table S1: Multipeak analysis of W 4f and S 2p photoemission lines for commercial WS_2_. Table S2: Multipeak analysis of W 4f and S 2p photoemission lines for Pristine 1T-WS_2_. Table S3: Multipeak analysis of W 4f and S 2p photoemission lines for 1T-WS_2_ deposited from B-1. Table S4: Multipeak analysis of W 4f and S 2p photoemission lines for 1T-WS_2_ deposited from MES 10 mM-pH 3. Table S5: Multipeak analysis of W 4f and S 2p photoemission lines for 1T-WS_2_ deposited from MES 10 mM-pH 5. Table S6: Multipeak analysis of W 4f and S 2p photoemission lines for 1T-WS_2_ deposited from MES 10 mM-pH 7. Table S7: Multipeak analysis of W 4f and S 2p photoemission lines for 1T-WS_2_ deposited from MES 10 mM-pH 8. Table S8: Multipeak analysis of W 4f and S 2p photoemission lines for 1T-WS_2_ deposited from PBS 10 mM-pH 7.4. Table S9: Multipeak analysis of W 4f and S 2p photoemission lines for 1T-WS_2_ deposited from PBS 1 mM-pH 7.4. Table S10: Multipeak analysis of W 4f and S 2p photoemission lines for 1T-WS_2_ deposited from PBS 0.1 mM-pH 7.4.
