# Supplementary material for: Electrophoretic Deposition of WS2 Flakes on Nanoholes Arrays—Role of Used Suspension Medium
Source: Materials (Basel). 2019 Oct 10;12(20):3286. doi: 10.3390/ma12203286 (PMC6829434; doi:10.3390/ma12203286)
Supplement: Supplementary file 1 [file materials-12-03286-s001.pdf]

materials

MDPI

Article – Supplementary Materials

Electrophoretic Deposition of WS_2 Flakes on Nanoholes Arrays—Role of Used Suspension Medium

Dario Mosconi [1], Giorgia Giovannini [2], Nicolò Maccaferri [3], Michele Serri [4], Stefano Agnoli [1] and Denis Garoli [4,*]

1 Dipartimento di Chimica, Università degli Studi di Padova, Via Marzolo 1, 35131 Padova, Italy
2 EMPA Federal Swiss research Institute, 9014 St. Gallen, Switzerland
3 Physics and Materials Science Research Unit, University of Luxembourg, L-1511 Luxembourg, Luxembourg
4 Istituto Italiano di Tecnologia, via Morego 30, 16163 Genova, Italy
* Correspondence: denis.garoli@iit.it;

Detailed XPS Analysis

Commercial 2H-WS_2 (before exfoliation)

Commercial WS_2 from Sigma-Aldrich was analyzed in order to get the reference for hexagonal 2H-phase BE and FWHM values, both for W 4f and S 2p lines. Moreover, since the surface oxidation due to air exposure generates WO_3, the values for this species was used as reference too.

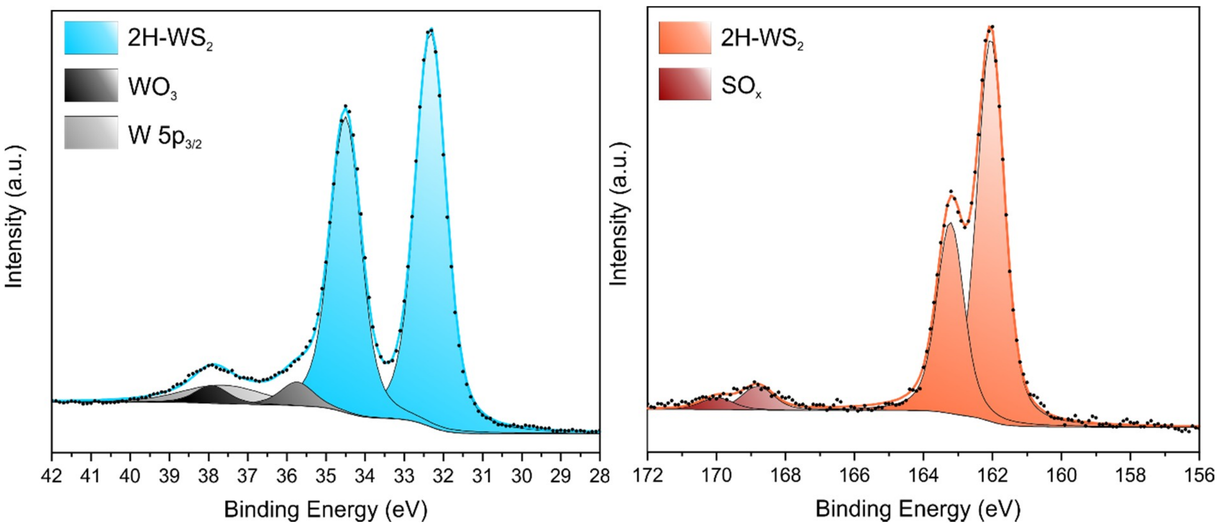

Figure S1. W 4f (left) and S 2p (right) core levels of commercial WS_2.

Table S1. Multipeak analysis of W 4f and S 2p photoemission lines for commercial WS_2.

W 4f			S 2p		
Species	BE (eV)	% at.	Species	BE (eV)	% at.
2H-WS₂	32.3	93.8	*2H-WS₂*	162.1	92.1
WO₃	35.7	6.2	SO_x	168.8	7.9

Pristine 1T-WS₂

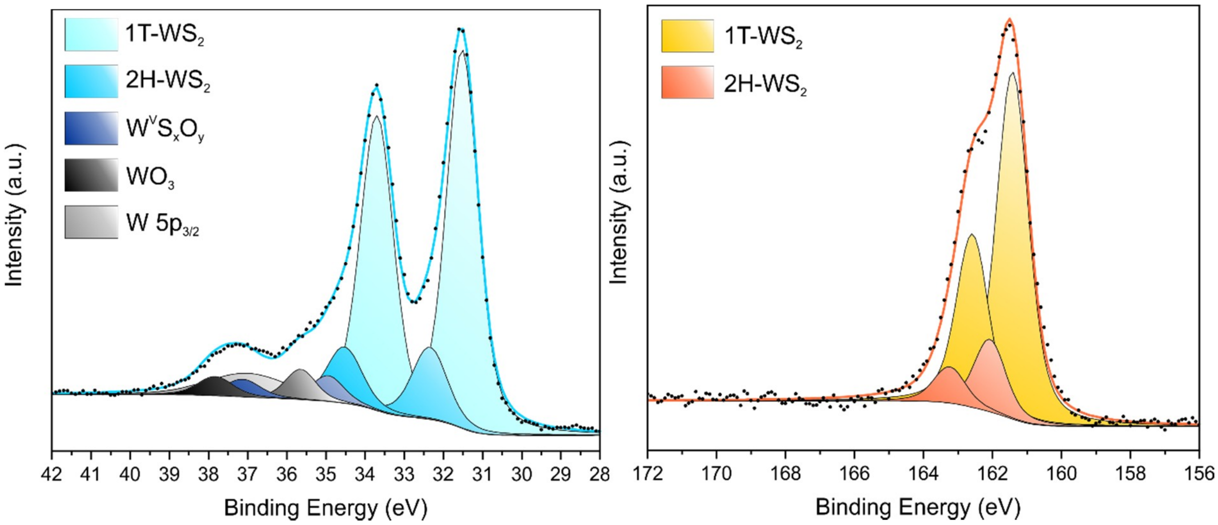

Figure S2. W 4f (left) and S 2p (right) core levels of Pristine 1T-WS_2.

Table S2. Multipeak analysis of W 4f and S 2p photoemission lines for Pristine 1T-WS_2.

W 4f			S 2p		
Species	BE (eV)	% at.	Species	BE (eV)	% at.
1T-WS₂	31.5	74.2	*1T-WS₂*	161.4	82.8
2H-WS₂	32.3	15.5	*2H-WS₂*	162.1	17.2
$W^VS_xO_y$	34.9	5.0	SO_x	-	-
WO₃	35.7	5.3			

1T-WS_2 deposited from B-1

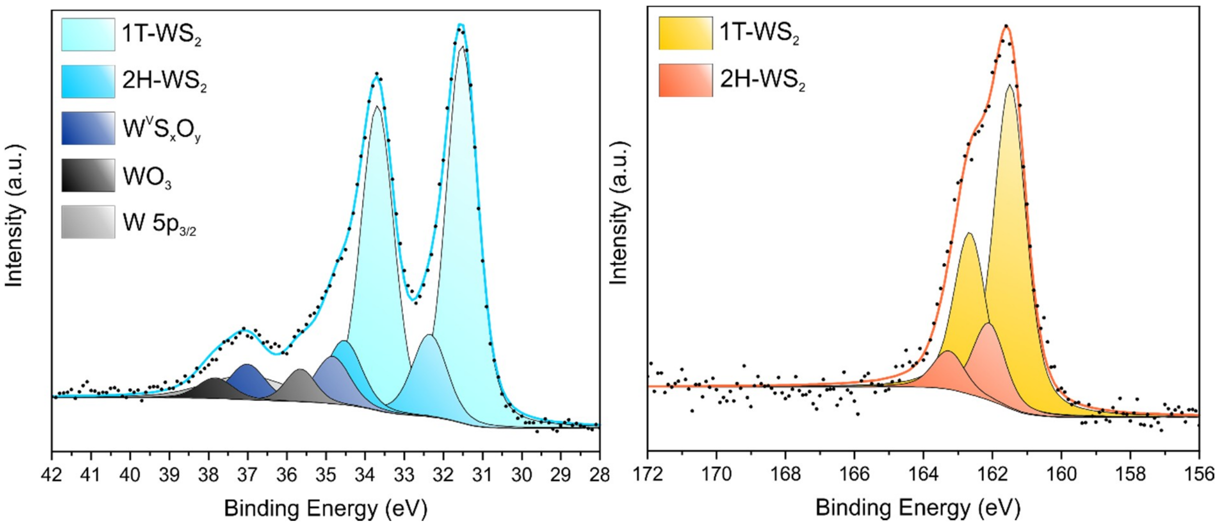

Figure S3. W 4f (left) and S 2p (right) core levels of 1T-WS_2 deposited from B-1.

Table S3. Multipeak analysis of W 4f and S 2p photoemission lines for 1T-WS_2 deposited from B-1.

W 4f			S 2p		
**Species**	**BE (eV)**	**% at.**	**Species**	**BE (eV)**	**% at.**
1T-WS_2	31.5	69.2	*1T-WS_2*	161.5	80.8
2H-WS_2	32.3	16.3	*2H-WS_2*	162.1	19.2
$W^{V}S_xO_y$	34.8	9.2	SO_x	-	-
WO_3	35.7	5.3			

1T-WS_2 deposited from MES 10 mM - pH 3

The presence of SO_x in S 2p line can be associated with adsorbed MES molecules.

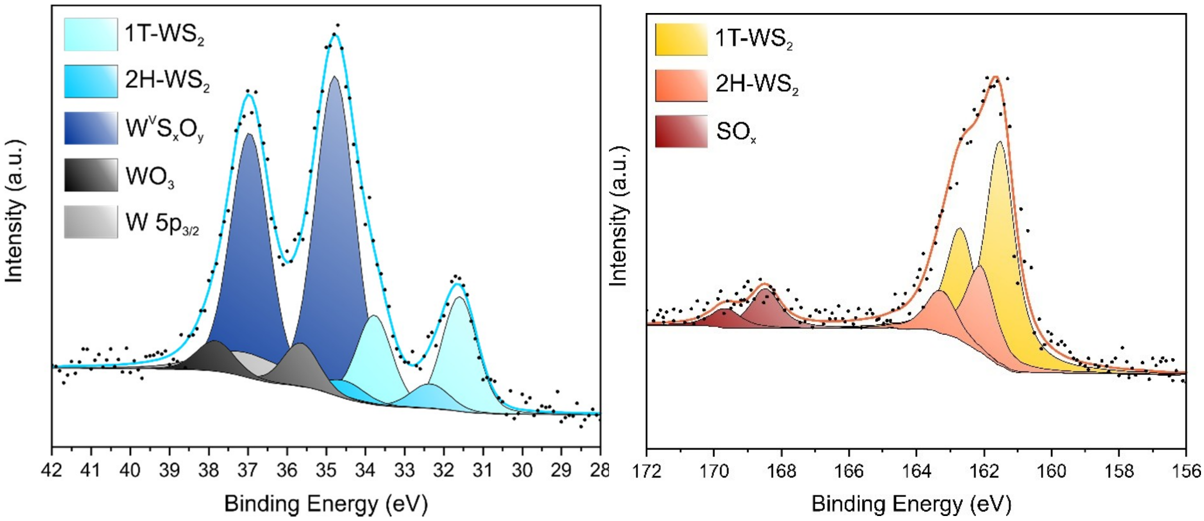

Figure S4. W 4f (left) and S 2p (right) core levels of 1T-WS_2 deposited from MES 10 mM - pH 3.

Table S4. Multipeak analysis of W 4f and S 2p photoemission lines for 1T-WS_2 deposited from MES 10 mM - pH 3.

W 4f			S 2p		
**Species**	**BE (eV)**	**% at.**	**Species**	**BE (eV)**	**% at.**
1T-WS_2	31.6	19.2	*1T-WS_2*	161.5	64.1
2H-WS_2	32.4	7.4	*2H-WS_2*	162.1	24.6
$W^VS_xO_y$	34.8	65.3	SO_x	168.4	11.3
WO_3	35.7	8.1			

1T-WS_2 deposited from MES 10 mM – pH 5

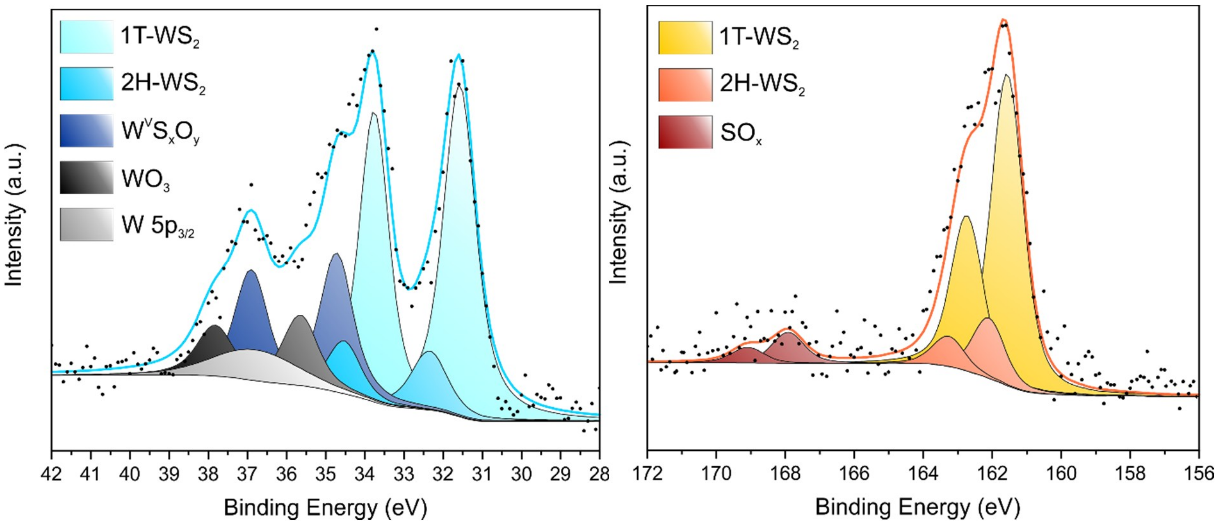

Figure S5. W 4f (left) and S 2p (right) core levels of 1T-WS_2 deposited from MES 10 mM - pH 5.

Table S5. Multipeak analysis of W 4f and S 2p photoemission lines for 1T-WS_2 deposited from MES 10 mM - pH 5.

W 4f			S 2p		
**Species**	**BE (eV)**	**% at.**	**Species**	**BE (eV)**	**% at.**
1T-WS_2	31.6	54.7	*1T-WS_2*	161.6	77.3
2H-WS_2	32.4	10.6	*2H-WS_2*	162.1	15.0
$W^VS_xO_y$	34.7	22.7	SO_x	167.2	7.7
WO_3	35.7	12.0			

1T-WS_2 deposited from MES 10 mM - pH 7

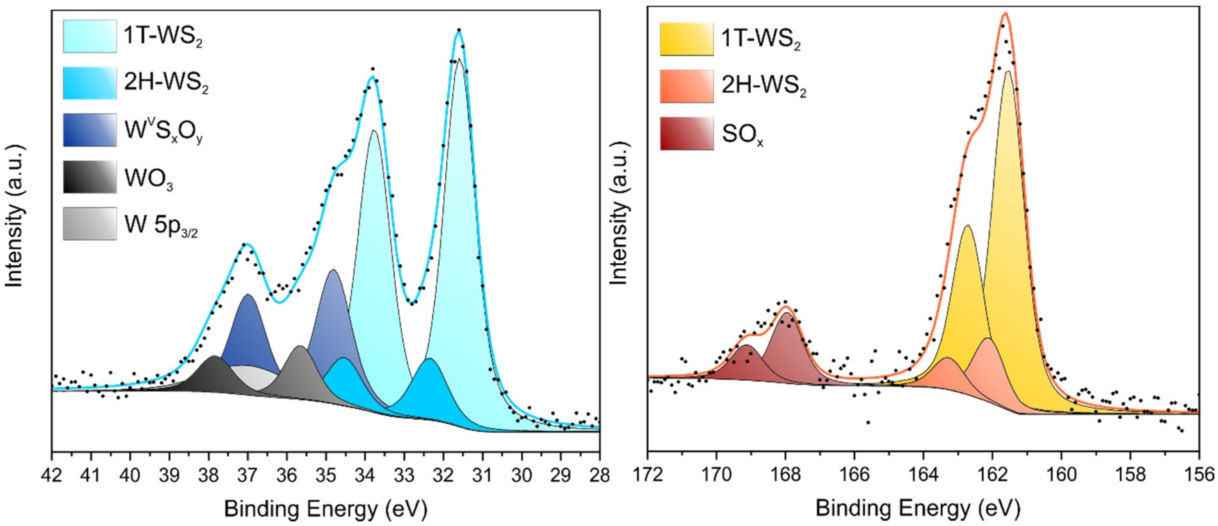

Figure S6. W 4f (left) and S 2p (right) core levels of 1T-WS_2 deposited from MES 10 mM - pH 7.

Table S6. Multipeak analysis of W 4f and S 2p photoemission lines for 1T-WS_2 deposited from MES 10 mM - pH 7.

W 4f			S 2p		
**Species**	**BE (eV)**	**% at.**	**Species**	**BE (eV)**	**% at.**
1T-WS_2	31.6	57.7	*1T-WS_2*	161.5	71.6
2H-WS_2	32.4	10.8	*2H-WS_2*	162.1	13.5
$W^VS_xO_y$	34.8	23.2	SO_x	168.0	14.9
WO_3	35.7	8.3			

1T-WS_2 deposited from MES 10 mM – pH 8

In this sample the SO_x band is particularly intense: due to basic pH, sulfonic acid is mainly in the deprotonated form, that may favor the adsorption on WS_2 sheets.

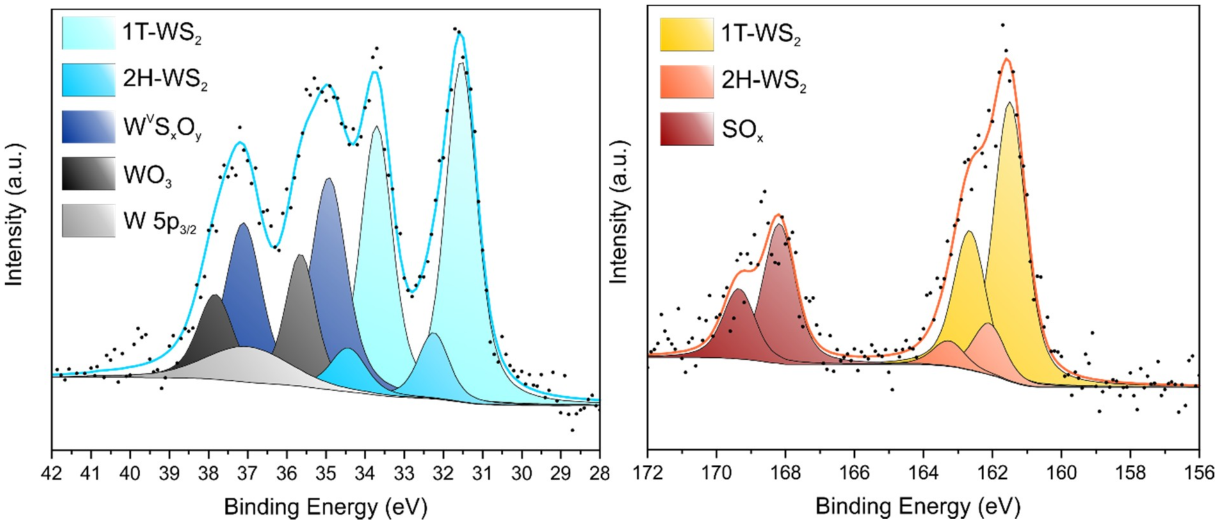

Figure S7. W 4f (left) and S 2p (right) core levels of 1T-WS_2 deposited from MES 10 mM – pH 8.

Table S7. Multipeak analysis of W 4f and S 2p photoemission lines for 1T-WS_2 deposited from MES 10 mM – pH 8.

W 4f			S 2p		
**Species**	**BE (eV)**	**% at.**	**Species**	**BE (eV)**	**% at.**
1T-WS_2	31.5	45.0	*1T-WS_2*	161.5	59.3
2H-WS_2	32.3	8.3	*2H-WS_2*	162.1	10.9
$W^VS_xO_y$	34.9	30.6	SO_x	168.2	29.8
WO_3	35.7	16.1			

$1T-WS_2$ deposited from PBS 10 mM – pH 7.4

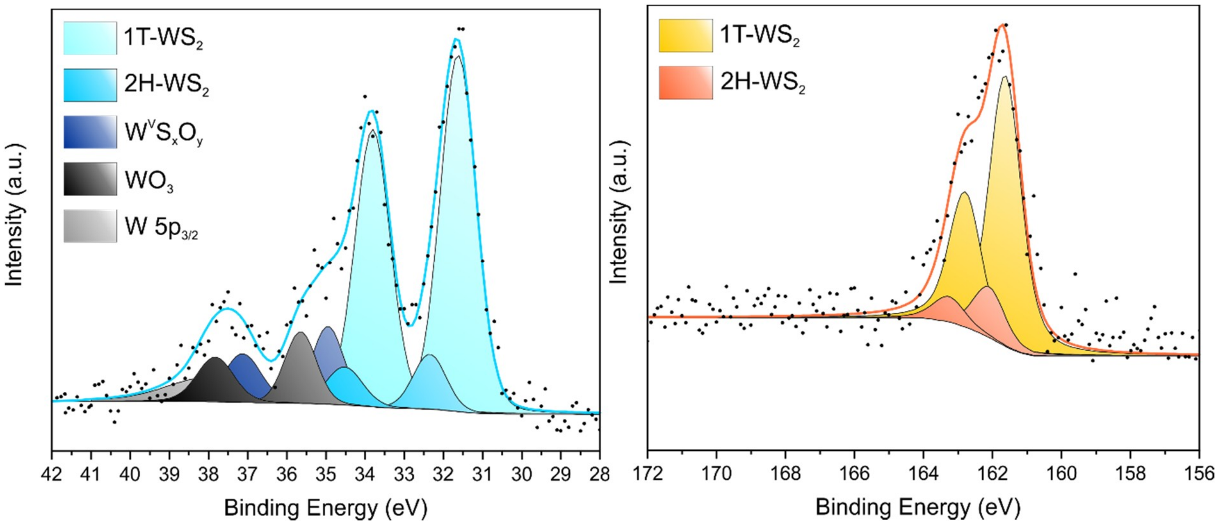

Figure S8. W 4f (left) and S 2p (right) core levels of $1T-WS_2$ deposited from PBS 10 mM – pH 7.4.

Table S8. Multipeak analysis of W 4f and S 2p photoemission lines for $1T-WS_2$ deposited from PBS 10 mM – pH 7.4.

W 4f			S 2p		
**Species**	**BE (eV)**	**% at.**	**Species**	**BE (eV)**	**% at.**
$1T-WS_2$	31.6	66.9	*$1T-WS_2$*	161.6	84.3
$2H-WS_2$	32.4	9.2	*$2H-WS_2$*	162.1	15.7
$W^VS_xO_y$	34.9	12.5	SO_x	-	-
WO_3	35.7	11.4			

1T-WS_2 deposited from PBS 1 mM – pH 7.4

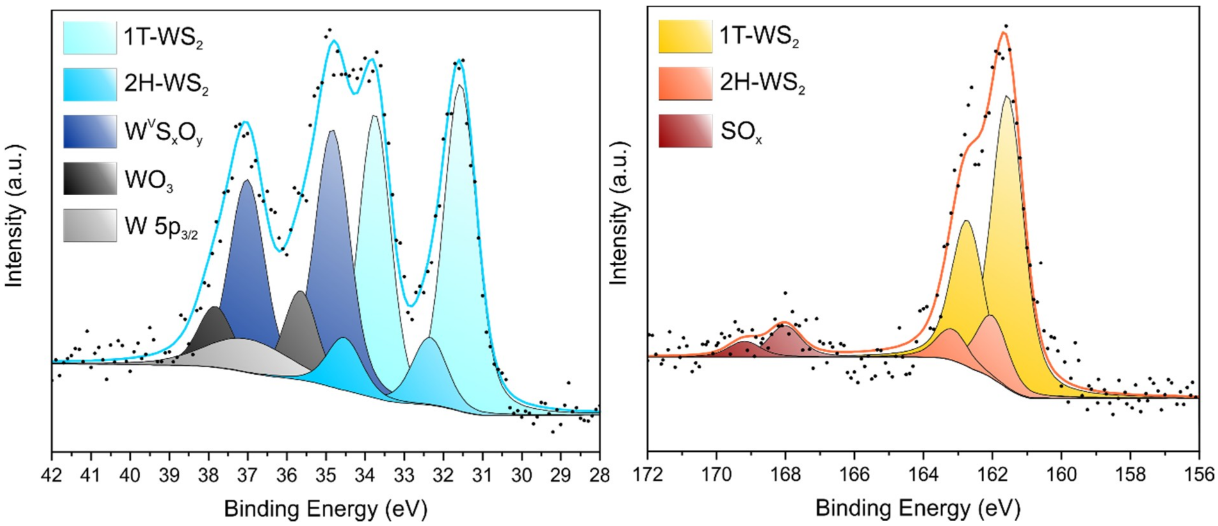

Figure S9. W 4f (left) and S 2p (right) core levels of 1T-WS_2 deposited from PBS 1 mM – pH 7.4.

Table S9. Multipeak analysis of W 4f and S 2p photoemission lines for 1T-WS_2 deposited from PBS 1 mM – pH 7.4.

	**W 4f**			**S 2p**	
**Species**	**BE (eV)**	**% at.**	**Species**	**BE (eV)**	**% at.**
1T-WS_2	31.6	44.1	*1T-WS_2*	161.5	75.7
2H-WS_2	32.4	9.4	*2H-WS_2*	162.1	16.2
$W^VS_xO_y$	34.8	35.4	SO_x	168.0	8.1
WO_3	35.7	11.1			

materials

1T-WS_2 deposited from PBS 0.1 mM – pH 7.4

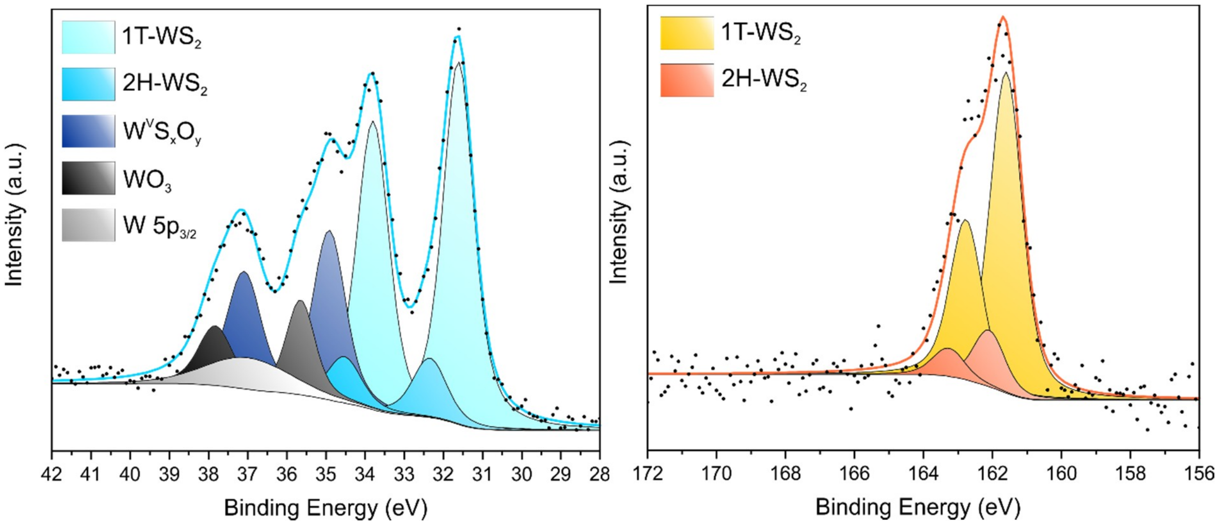

Figure S10. W 4f (left) and S 2p (right) core levels of 1T-WS_2 deposited from PBS 0.1 mM – pH 7.4.

Table S10. Multipeak analysis of W 4f and S 2p photoemission lines for 1T-WS_2 deposited from PBS 0.1 mM – pH 7.4.

W 4f			S 2p		
**Species**	**BE (eV)**	**% at.**	**Species**	**BE (eV)**	**% at.**
1T-WS_2	31.6	53.6	*1T-WS_2*	161.5	85.1
2H-WS_2	32.4	9.4	*2H-WS_2*	162.1	14.9
$W^VS_xO_y$	34.9	24.5	SO_x	-	-
WO_3	35.7	12.4			
